# Abnormal peripheral blood cell counts in neurofibromatosis type 1

**DOI:** 10.1038/s41598-022-23739-z

**Published:** 2022-11-05

**Authors:** Yoshimasa Nobeyama, Ken-ichi Yasuda, Akihiko Asahina

**Affiliations:** Department of Dermatology, The Jikei University School of Medicine, 25-8 Nishi-Shimbashi 3-chome, Minato-ku, Tokyo, 105-8461 Japan

**Keywords:** Medical research, Signs and symptoms

## Abstract

Neurofibromatosis type 1 (NF1), also known as von Recklinghausen disease, is an autosomal dominant disease characterized by neurofibromas with infiltration of mast cells. Neutrophil-to-lymphocyte ratio (NLR), lymphocyte-to-monocyte ratio (LMR), platelet-to-lymphocyte ratio (PLR) and basophil-to-lymphocyte ratio (BLR) are examined as markers for various diseases. However, these parameters have not yet been assessed for NF1. This study therefore examined these parameters in NF1 patients. We recruited 153 NF patients (78 males, 75 females) and 51 control patients (31 males, 20 females). Complete blood counts were performed, then NLR, LMR, PLR and BLR were calculated. Neutrophil count was significantly higher in male NF1 patients than in male controls. Lymphocyte count was significantly lower in NF1 patients than in controls for both sexes. Monocyte count was significantly higher in male NF1 patients than in male controls. Basophil count was significantly higher in male NF1 patients than in male controls. NLR, PLR and BLR were significantly higher in NF1 patients than in controls for both sexes. LMR was significantly lower in NF1 patients than in controls for both sexes. NF1 shows high NLR, PLR and BLR and low lymphocyte count and LMR.

## Introduction

Neurofibromatosis type 1 (NF1), also called von Recklinghausen disease, is an autosomal dominant disease caused by mutations in the *NF1* gene encoding neurofibromin that occurs in approximately 1 in 3000 people with 100% penetrance, but highly variable expressivity^1–3^. Neurofibromin is a GTPase-activating protein that negatively regulates the RAS/mitogen-activated protein kinase (MAPK) pathway by accelerating the hydrolysis of RAS-bound GTP^2,3^. Therefore, the loss of neurofibromin results in cell growth and proliferation. The symptoms and signs of the disease include café-au-lait spots, cutaneous neurofibromas, plexiform neurofibromas, vasculopathy, optic nerve and central nervous system gliomas, learning difficulties, malignant peripheral nerve sheath tumors, scoliosis, tibial dysplasia, gastrointestinal stromal tumors, and lung disorders^1–4^.

Some evidence suggests that the development of several clinical manifestations of NF1 is associated with inflammation. Staser et al. suggested that the inflammation caused by Nf1-haploinsufficient mast cells accelerates tumor formation and growth by mediating key mitogenic signals in mouse models of plexiform neurofibroma^5^. Liao et al. reported that the development of neurofibromas is associated with both macrophages and mast cells^6^. Lasater et al. demonstrated that increases in inflammatory cells and cytokines were linked to vascular inflammation and vaso-occlusive disease in NF1 patients without overt vascular disease^7^. Clinical manifestations of NF1 are thus likely associated with inflammation, or with inflammatory cells at the very least.

Many researchers suggest clinical usefulness of the ratios of differential white blood cell counts, which are obtained from the complete blood count (CBC) test, a simple, economic and widely used basic hematological test. For example, neutrophil-to-lymphocyte ratio (NLR) is regarded as a useful marker for estimating inflammation in Behçet's disease^8^, as well as predicting the prognosis of various diseases including malignancies, peripheral vascular diseases and cardiovascular events^9–13^. Lymphocyte-to-monocyte ratio (LMR) has been reported as useful for predicting the prognosis of acute ischemic stroke and colorectal carcinoma^14,15^. Platelet-to-lymphocyte ratio (PLR) has been reported to be useful for examining inflammation of rheumatic diseases^16^, as well as predicting the prognosis of urothelial carcinoma and colorectal carcinoma^17,18^. Basophil-to-lymphocyte ratio (BLR) has been reported to show abnormal levels in systemic autoimmune rheumatic disease^19^. NLR, LMR, PLR and BLR thus potentially represent the pathogenesis and conditions of tumors and inflammatory diseases.

Misirlioglu et al. examined NLR, LMR and PLR in 82 patients with a neurofibroma, 79 patients with a schwannoma, and 20 with a malignant peripheral nerve sheath tumor (MPNST). Consequently, neurofibroma, schwannoma, and MNPST groups had significantly higher median NLR, compared to the control group, while the median LMR was significantly lower in these groups. However, the median PLR was higher only in the MPNST group, compared to the control group (p < 0.001)^20^. The study did not indicate whether the examined patients were affected with NF1, although the development of several clinical manifestations of NF1 is suggested to be related to inflammation.

This study analyzed the results of CBC testing, including NLR, LMR, PLR and BLR, in NF1 patients and control patients. We consequently detected characteristic findings from peripheral blood cell counts in NF1 patients.

## Patients and methods

### Patients and assessments

The ethics committee of The Jikei University School of Medicine, Tokyo, Japan, approved the study protocol, and informed consent was obtained in the form of opt-out for all patients. This study was conducted in accordance with the ethical principles of the declaration of Helsinki.

We recruited 153 patients with NF1 (78 males, 75 females) and 51 non-NF1 patients suffering from lipoma (31 males, 20 females), who met the following inclusion criteria: (i) referral to our hospital; (ii) for NF1 patients, fulfilment of the diagnostic criteria from the National Institutes of Health Consensus Development Conference in 1987^21^; (iii) surgical excision of neurofibroma or lipoma; (iv) results available from blood testing within 6 weeks preoperatively; and (v) no acute diseases evident at the preoperative check or intraoperatively.

CBC testing was performed within 6 weeks preoperatively using an automatic blood cell count analyzer (XE-5000; Sysmex, Kobe, Japan). With this analyzer, normal ranges are defined as follows: white blood cell count, 3300–8600/μL; neutrophil count, 1300–5700/μL; lymphocyte count, 1000–3000/μL; monocyte count, 200–600/μL; eosinophil count, 100–300/μL; basophil count, 0–100/μL; and platelet count, 158–358/nL.

### Statistical analysis

Statistical analysis was performed using commercially available software (SPSS version 22; SPSS Japan, Tokyo, Japan). The Mann–Whitney U test was performed to test differences in values between NF1 patients and lipoma patients as controls. Values of *P* < 0.05 were considered statistically significant.

### Ethics approval and consent to participate

The ethics committee of The Jikei University School of Medicine, Tokyo, Japan, approved the study protocol, and informed consent was obtained in the form of opt-out for all patients. This study was conducted in accordance with the ethical principles of the declaration of Helsinki.

## Results

### Assessment of patients with lipoma as normal controls

Mean (± standard deviation) ages of patients with NFl and those with lipoma were 51.34 ± 10.85 years and 52.91 ± 11.18 years in males and 52.35 ± 11.00 years and 53.00 ± 12.39 years in females, respectively. Between patients with NF1 and those with lipoma, statistical analyses revealed no significant difference in either sex (*P* = 0.229 and 0.756 in male and female patients, respectively).

Mean counts in male and female patients with lipoma were as follows: white blood cell count, 6303/μL and 6165/μL; neutrophil count, 3586/μL and 3573/μL; lymphocyte count, 2083/μL and 2056/μL; monocyte count, 398/μL and 275/μL; eosinophil count, 211/μL and 212/μL; basophil count, 25/μL and 41/μL; and platelet count, 252/nL and 248/nL, respectively (Table 1). These values are indicated within normal ranges by wide margins. Based on these findings, we regarded these data as normal controls compared to the analyses of data from NF1 patients.

**Table 1:** Comparison of differential white blood cell counts between NF1 patients and controls.

	Mean value ± standard deviation			
	Sex	NF1	Control	*p*-value
White blood cells	Male	6065 ± 1203	6303 ± 1706	0.229
	Female	6208 ± 1907	6165 ± 2204	0.756
Neutrophils	Male	3971 ± 1080	3586 ± 1223	0.011
	Female	4251 ± 1543	3573 ± 2034	0.063
Lymphocytes	Male	1430 ± 348	2083 ± 548	< 0.001
	Female	1376 ± 437	2056 ± 554	< 0.001
Monocytes	Male	458 ± 398	398 ± 148	0.001
	Female	390 ± 170	275 ± 108	0.301
Eosinophils	Male	213 ± 164	211 ± 158	0.987
	Female	169 ± 226	212 ± 191	0.258
Basophils	Male	44 ± 28	25 ± 13	< 0.001
	Female	41 ± 21	41 ± 24	0.798
Platelets	Male	252 ± 50	252 ± 50	0.917
	Female	271 ± 77	248 ± 59	0.203

### Counts for each type of white blood cell in NF1 patients and controls

Mean values and standard deviations of white blood cell count, neutrophil count, lymphocyte count, monocyte count, eosinophil count, basophil count and platelet count of NF1 patients were compared with controls (Table 1). Mean white blood cell counts of NF1 patients vs. those of controls were 6065/μL vs. 6303/μL in males and 6208/μL vs. 6165/μL in females. Mean neutrophil counts were 3971/μL vs. 3586/μL in males and 4251/μL vs. 3573/μL in females. Mean lymphocyte counts were 1430/μL vs. 2083/μL in males and 1376/μL vs. 2056/μL in females. Mean monocyte counts were 458/μL vs. 398/μL in males and 390/μL vs. 275/μL in females. Mean eosinophil counts were 213/μL vs. 211/μL in males and 169/μL vs. 212/μL in females. Mean basophil counts were 44/μL vs. 25/μL in males and 41/μL vs. 41/μL in females. Mean platelet counts were 252/nL vs. 252/nL in males and 271/nL vs. 248/nL in females.

Between NF1 patients and controls, statistical analyses revealed that: (i) white blood cell counts did not differ significantly in either sex; (ii) neutrophil count was significantly higher in NF1 patients than in controls in males, but not in females; (iii) lymphocyte count was significantly lower in NF1 patients than in controls for both sexes; (iv) monocyte count was significantly higher in NF1 patients than in controls in males, but not in females; (v) eosinophil count did not differ significantly in each sexes; (vi) basophil count was significantly higher in NF1 patients than in controls in males, but not in females; and (vii) platelet count did not differ significantly in either sex (Table 1).

### NLR, LMR, PLR and BLR in NF1 patients and controls

Since the results of comparisons of absolute counts for each type of white blood cell between NF1 patients and controls differed between sexes, except for the lymphocyte count, we focused on NLR, LMR, PLR and BLR (Supplementary Fig. 1). Mean NLR, LMR, PLR and BLR of NF1 patients were compared to those of normal controls (Table 2). Mean NLR of NF1 patients compared to controls was 2.96 vs. 1.76 in males and 3.24 vs. 1.84 in females. Mean LMR of NF1 patients compared to controls was 3.69 vs. 5.52 in males and 3.83 vs. 9.36 in females. Mean PLR of NF1 patients compared to controls was 184.68 vs. 127.66 in males and 207.69 vs. 128.82 in females. Mean BLR of NF1 patients compared to controls was 32.06 vs. 13.08 in males and 31.79 vs. 21.16 in females.

**Table 2:** Comparison of NLR, LMR, PLR and BLR between NF1 patients and controls.

	Mean value ± standard deviation			
	Sex	NF1	Control	*p*-value
NLR	Male	2.96 ± 1.10	1.76 ± 0.50	< 0.001
	Female	3.24 ± 1.17	1.84 ± 1.09	< 0.001
LMR	Male	3.69 ± 1.63	5.52 ± 1.40	< 0.001
	Female	3.83 ± 1.33	9.36 ± 6.88	< 0.001
PLR	Male	184.68 ± 49.29	127.66 ± 34.38	< 0.001
	Female	207.69 ± 61.73	128.82 ± 44.00	< 0.001
BLR	Male	32.06 ± 19.57	13.08 ± 9.11	< 0.001
	Female	31.79 ± 16.39	21.16 ± 14.85	< 0.001

Statistical analyses revealed that NLR, PLR and BLR were significantly higher in NF1 patients than in controls for both sexes, and LMR was significantly lower in NF1 patients than in controls for both sexes (Table 2).

## Discussion

This study offers the first evidence that NLR, PLR and BLR are higher, and lymphocyte count and LMR are lower in NF1 patients than in non-NF1 individuals.

Many reports in the literature have described that high NLR and PLR are associated with the pathogenesis and clinical course of cancers^9,10,18,22^, compatible with several reports that neutrophils play various oncogenic roles, including tumor growth, angiogenesis, metastasis and T-cell suppression^22,23^, while lymphocytes potentially act as tumor-suppressive immune responders^24^. Considering that high NLR and PLR also indicate inflammation^8,16^, the high ratios in NF1 are suggested to represent tumorigenesis of neurofibroma through inflammation, mainly associated with mast cells located in neurofibroma^5–7^. Because the inflammation localized in neurofibromas may not sufficiently stimulate signals for the production of common inflammation markers such as C-reactive protein, the assessment of inflammation by CBC testing in NF1 may have rarely been a focus before now.

We also focused on BLR for NF1, because: (i) mast cells infiltrate into neurofibromas; and ii) mast cells and basophils share functional similarities, including long-term inflammatory or immunologic responses as well as immediate hypersensitivity reactions^25^. This study clearly showed that BLR was higher in NF1 patients than in controls. In contrast, Yang et al. demonstrated a significantly low BLR in systemic autoimmune rheumatic diseases^19^. These facts led to three considerations. First, BLR should not be regarded as a universal inflammatory marker. Second, a high BLR may be somewhat specific for NF1. Third, reference to BLR may be difficult in NF1 patients with complicating systemic autoimmune rheumatic diseases. On the other hand, basophils and mast cells exhibit fundamental differences^25^. Basophils do not proliferate after terminal differentiation and the cells circulate in the peripheral blood. In contrast, mast cells reside in peripheral tissue and retain the ability to proliferate. In NF1, the association between basophils and mast cells should be further investigated.

The application of these parameters to clinical practice may be premature because the results obtained did not reveal relationships with clinical symptoms, including the tumor burden, skeletal abnormalities, vasculopathy, and learning difficulties. Therefore, further studies are warranted to identify the symptoms that affect these parameters and also the extent of their effects.

NF1 is an RASopathy, a comprehensive pathological concept covering genetic syndromes caused by germline mutations in the genes that encode the molecules associated with the RAS/MAPK pathway^26^. In addition to NF1, RASopathy includes Noonan syndrome, Noonan syndrome with multiple lentigines, Costello syndrome, cardio-facio-cutaneous syndrome, and Legius syndrome^26^. Whether these other RASopathies also show high NLR, PLR and BLR and low lymphocyte count and LMR is an interesting question worth exploring.

Lee et al. calculated mean NLR, LMR and PLR across all ages from 12,160 blood samples collected from healthy patients in South Korea^27^. Our data may be comparable with the data described by Lee et al., because patients included in our data were uniformly Japanese, representing an East Asian ethnicity similar to South Koreans. NLR for our lipoma patients compared to the data from Lee et al. showed 1.76 vs. 1.63 for males and 1.84 vs. 1.66 for females. Similarly, our data compared to theirs indicated LMR of 5.52 vs. 5.05 for males and 9.36 vs. 5.60 for females, and PLR of 127.66 vs. 122.75 for males and 128.82 vs. 142.76 for females. All parameters calculated from our lipoma patients were broadly compatible with those calculated from healthy South Koreans, with the exception of LMR in females. This information supports the notion that lipoma patients can largely be regarded as healthy controls in terms of these comparisons.

Some limitations to this study need to be considered. First, data were lacking for other inflammatory markers such as C-reactive protein and erythrocyte sedimentation rate. Therefore, whether the NF1 patient group included NF1 patients with acute inflammatory diseases causing high NLR and/or PLR remains unclear. Second, an assessment of the clinical severity of NF1 was not performed. It currently remains unclear whether the parameters examined in the present study are associated with the clinical severity of NF1, including the tumor burden. Third, data were lacking in terms of complications in NF1 patients and control patients, such as systemic inflammatory diseases, cardiovascular diseases, diabetes mellitus and malignancies. NLR, LMR, PLR and/or BLR may be affected by patient status in terms of systemic inflammatory disease, risk of cardiovascular events, control of type 2 diabetes mellitus or the presence of malignancy^9,12,19,22,28,29^. Fourth, the present results lead only to speculations that the mechanisms of inflammation may be associated with NF1. Therefore, further studies are needed to show direct evidence for a relationship between the mechanisms of inflammation and NF1.

In conclusion, this study demonstrated that NF1 patients show high NLR, PLR and BLR and low lymphocyte count and LMR. These parameters may represent the inflammation associated with neurofibroma tumorigenesis in NF1.

## Supplementary Information

Supplementary Figure 1.

## Data Availability

The data that support the findings of this study are available from the corresponding author, Y.N., upon reasonable request.
