# Supplementary figures and images for: Abnormal peripheral blood cell counts in neurofibromatosis type 1
Source: Sci Rep. 2022 Nov 5;12:18800. doi: 10.1038/s41598-022-23739-z (PMC9637152; doi:10.1038/s41598-022-23739-z)

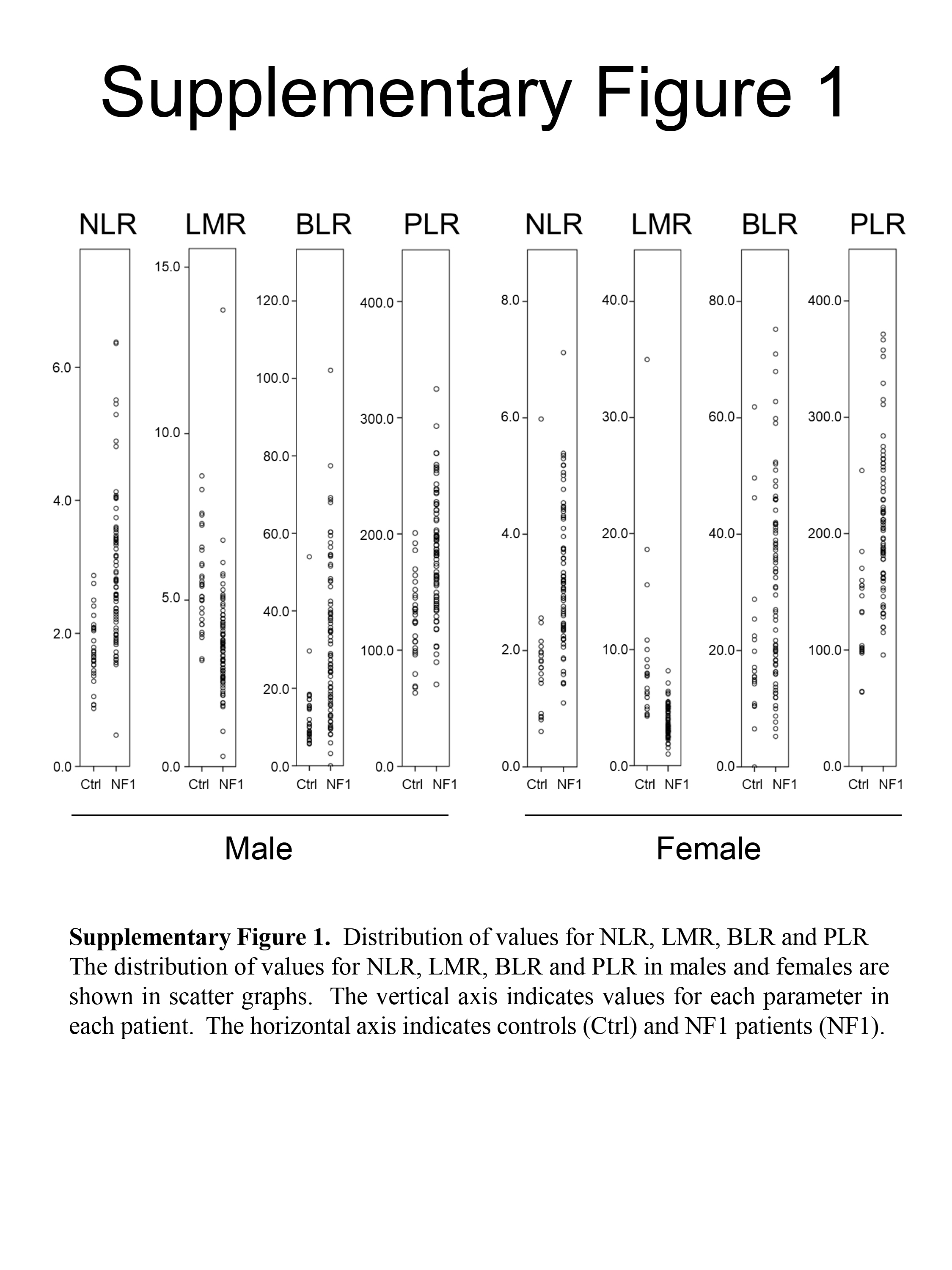

Supplement: Supplementary file 1 — Supplementary Figure 1. [file 41598_2022_23739_MOESM1_ESM.jpg]
